# Methods to Assess Adult and Adolescent Patients’ Adherence to Antimalarial Treatment: A Systematic Review

**DOI:** 10.3389/fphar.2022.796027

**Published:** 2022-04-27

**Authors:** Heloísa Ferreira Pinto Santos, Lusiele Guaraldo, Renata Saraiva Pedro, Luana Santana Damasceno, Cláudio Tadeu Daniel-Ribeiro, Patrícia Brasil

**Affiliations:** ^1^ Clinical Research Laboratory on Acute Febrile Illnesses, Instituto Nacional de Infectologia Evandro Chagas, Fundação Oswaldo Cruz (Fiocruz), Rio de Janeiro, Brazil; ^2^ Clinical Advice, Instituto de Tecnologia em Imunobiológicos, Fiocruz, Rio de Janeiro, Brazil; ^3^ Malaria Research Laboratory, Instituto Oswaldo Cruz, Fiocruz, Rio de Janeiro, Brazil; ^4^ Centro de Pesquisa, Diagnóstico e Treinamento em Malária, Fiocruz and Secretaria de Vigilância em Saúde, Ministério da Saúde, Rio de Janeiro, Brazil

**Keywords:** malaria, adherence, compliance, persistance, antimalarial treatment, method, systematic review

## Abstract

Malaria is a curable disease for which early diagnosis and treatment, together with the elimination of vectors, are the principal control tools. Non-adherence to antimalarial treatment may contribute to therapeutic failure, development of antimalarial resistance, introduction or resurgence of malaria in non-endemic areas, and increased healthcare costs. The literature describes several methods to directly or indirectly assess adherence to treatment, but no gold standard exists. The main purpose of this review is to systematize the methods used to assess patient adherence to antimalarial treatment. A systematic review was performed, in accordance with the PRISMA statement, of the following databases: LILACS, EMBASE, PUBMED, COCHRANE, GOOGLE SCHOLAR, WEB OF SCIENCE, SCOPUS, and OPENGREY, through 14 December 2021. A snowball search was also performed by screening the references of the included studies as well as those cited in relevant reviews. Inclusion criteria were reporting assessment of the patient’s adherence to antimalarials in individuals with laboratory diagnosis of malaria, the description of antimalarials prescribed, and adherence estimates. Exclusion criteria were studies exclusively about directly observed therapy, studies of populations ≤12 yo and guidelines, commentaries, reviews, letters, or editorials. Study quality was assessed using MINORS and the Cochrane Risk of Bias Tool. Proportions were calculated to measure frequencies considering the number of articles as the denominator. Twenty-one studies were included in this review. Most of them (76.5%) assessed adherence to *falciparum* malaria treatment. Seventeen studies (80.9%) used a combination of methods. The methods described were pill counts, self-reports, biological assays, use of electronic pillboxes, and clinical cure. It was possible to identify different adherence classifications for all the methods used. Our review found that indirect methods like pill counts and self-reports are the most commonly used. Combining an method that gives solid proof of the ingestion of medication and a method that completes the research with information regarding factors, beliefs or barrier of adherence seems to be the best approach. Future studies of antimalarial treatment should standardize adherence classifications, and collect data on the types and causes of nonadherence, which can contribute to the development of tools to promote medication adherence.

**Systematic Review Registration:** https://www.crd.york.ac.uk/prospero/display_record.php?ID=CRD42020148054, identifier CRD42020148054

## Introduction

Malaria is a treatable disease endemic in several countries of Africa, Asia, and South America. The World Health Organization (WHO) estimates (World Health Organization, 2020) that 229 million cases and 409,000 deaths occurred worldwide in 2019. Prompt diagnosis and treatment are the principal tools for the control of malaria. WHO recommendations for antimalarial treatment vary according to the species responsible for the infection: artemisinin-based combination therapy (ACT) for uncomplicated *falciparum* malaria; chloroquine plus primaquine for *Plasmodium vivax* or *P. ovale*, and chloroquine for *P. malarie* (World Health Organization and Global Malaria Programme, 2015).

Non-adherence to antimalarial treatment is thought to be one of the main causes of failure and may contribute to the maintenance of malaria transmission in a given area, development of antimalarial resistance, inadequate control of the disease, and increased healthcare costs (Duarte and Gyorkos, 2003; World Health Organization and Global Malaria Programme, 2015).

Medication adherence, defined by WHO as “the extent to which a person’s behavior—taking medication, following a diet, and/or executing lifestyle changes—corresponds with agreed recommendations from a health care provider”, is a multidimensional phenomenon determined by the interaction of factors such as access to medication, patient behavior, socio-economic status, the pathology of the disease, and the treatment complexity (Sabate, 2003). Methods for measuring medication adherence are classified as direct or indirect (Cramer, 1991). Direct methods are biological assays that measure the concentrations of drugs, metabolites, or biomarkers in blood, hair, or urine. Indirect methods include self-reports (interviews and questionnaires), medication measurement (pill count), and electronic monitoring devices (Medication Event Monitoring System, MEMS), which record the opening of a medicine bottle.

Adherence to antimalarials has been reviewed previously, with focus on the effectiveness of interventions to improve adherence and effects on therapeutic response (Yeung and White, 2005), patterns of adherence and associated factors (Bruxvoort et al., 2014; Ahluwalia et al., 2020) on ACT exclusively (Banek et al., 2014; Yakasai et al., 2015). To date, no method for measuring medication adherence has been validated for malaria treatment. The present review aimed to systematize the information about the methods used to assess adherence to antimalarial therapy.

## Materials and Methods

### Search

This review was developed according to the recommendations of the PRISMA statement (Page et al., 2021), and the protocol was registered in international prospective register of systematic reviews (PROSPERO, CRD42020148054) (Santos et al., 2020). A systematic search for the identification of studies about measurement of adherence to antimalarials was conducted through 14 December 2021 in the following databases: LILACS, EMBASE, MEDLINE (Medical Literature Analysis and Retrieval System Online, interface PUBMED), COCHRANE, GOOGLE SCHOLAR, WEB OF SCIENCE, SCOPUS, and OPENGREY. Additionally, a snowball search was performed by screening the references of the studies included in this review for eligibility, as well as references of other reviews (Yeung and White, 2005; Banek et al., 2014; Bruxvoort et al., 2014; Yakasai et al., 2015).

The search queries were developed using the PECO strategy. The PECO of this review is: P: participants with a laboratory diagnosis of malaria at least 13 years of age; E: malaria treatment; C: Not applicable; O: Methods to assess adherence to treatment. The search descriptors used were malaria, treatment, drug therapy, antimalarials, adherence (medication, patient), compliance (medication, patient) and humans. The search strategy was adapted to each database as necessary. The complete search strategy is available (Supplementary Table S1). There were no language or year restrictions on the searches of the databases.

### Selection

References were imported to the reference manager Zotero (Zotero, 2020) and duplicates were removed. The selection was performed by pairs of independent reviewers (HFPS and RSP, HFPS and LSD) using the web application Rayyan (Ouzzani et al., 2016). Studies were included if reporting assessment of patients’ adherence to antimalarials in individuals with laboratory diagnosis of malaria, adherence estimates, and the antimalarials prescribed. We excluded studies exclusively about directly observed therapy (DOT) and studies of populations ≤12 years old. DOT studies were excluded as they do not assess but ensure adherence. Children depends on their parents or caregivers to administer their medications, making the evaluation of adherence more complex (Santer et al., 2014; El-Rachidi et al., 2017). Guidelines, commentaries, reviews, letters, and editorials were also excluded. Titles and abstracts were screened for relevance then full text reading was performed. Discrepancies were reviewed and resolved by consensus between two other reviewers (LG and PB).

### Data Extraction and Quality Assessment

Data were extracted independently by the same pairs reviewers who selected the studies (HFPS and RSP, HFPS and LSD). Discrepancies were reviewed and resolved by consensus. A standardized data extraction form was developed for the review using the software Epidata v. 3.1, including the following sections: identification of the study (authors, journal, year of publication, and language); study characteristics (design and duration); study population (total number of patients, age, sex, inclusion of pregnant women, and the plasmodial species responsible for the infection); treatment prescribed (drugs and duration of treatment); assessment of the patient’s adherence (method for measuring patient adherence to treatment, adherence classification, adherence criteria, estimate of adherence thereby obtained); and other miscellaneous information such as factors posited to explain nonadherence, and any caveats that the authors made about the estimates of adherence or limitations of the study.

Study quality was assessed using the Methodological Index for Non-randomized Studies (MINORS) for observational studies (Slim et al., 2003) and the Cochrane Risk of Bias Tool for Randomized Controlled Trials for clinical trials (Higgins and Green, 2011). Two reviewers (HFPS and LG) evaluated each article independently and discrepancies were resolved by consensus.

### Data Synthesis and Analysis

A description of the studies regarding the year of publication, study design, population (country, sample size, sex, and age), type of infection, antimalarial treatment described with treatment duration, adherence assessment methods (description, assessment day, and adherence categories) and the resulting estimates were performed. Proportions were calculated to measure frequencies of variables considering the number of articles as the denominator.

## Results

**FIGURE 1:**
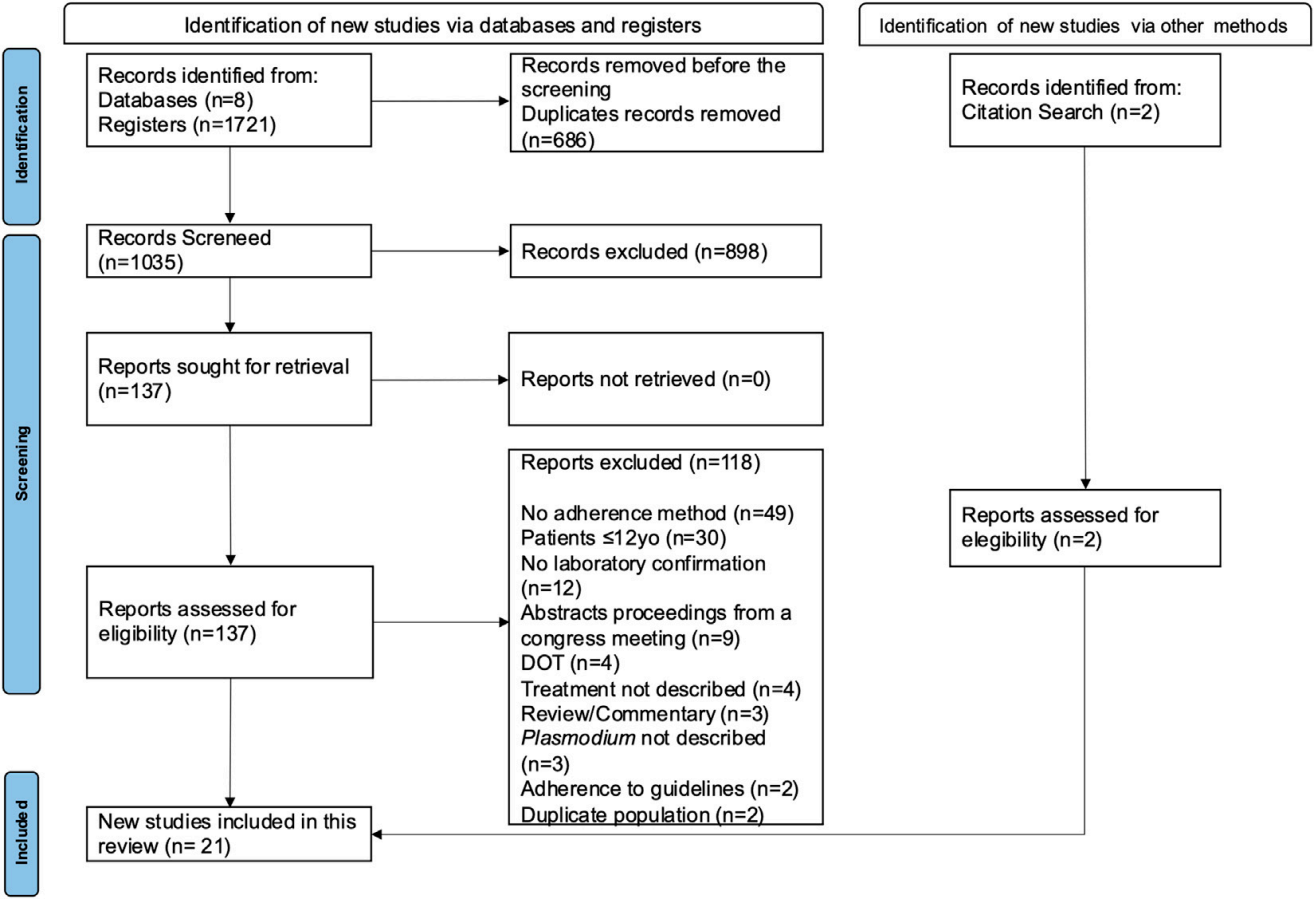
Flowchart of systematic search and selection for adherence methods to antimalarial studies.

The search strategy returned 1721 studies (Figure 1). After the exclusion of duplicates and application of inclusion criteria, 19 studies were deemed suitable for inclusion in this review. Two additional studies were included after snowball search. Thus, a total of 21 studies were selected for this review.

Characteristics of the included studies (country, design, population, infection/treatment, adherence methods, estimates and quality) are outlined in Supplementary Table S2. The included studies were published between 1997 and 2020, and 66.7% (14/21) were published after 2011. They were carried out in countries that included malaria-endemic areas in Africa, Asia, and the Americas. In the Americas, all studies were conducted in Brazil. Regarding study design of the 21 studies included in this review, 14 (66.7%) were observational, six (28.5%) experimental, and one quasi-experimental (4.8%). The sample size described in the studies varied from 27 to 300 participants for the observational/quasi-experimental studies and from 50 to 324 participants for the experimental ones. The patient populations were children and adults, and nine (42.9%) studies excluded pregnant women (Fungladda et al., 1998; Lemma et al., 2011; Ferreira et al., 2014; Minzi et al., 2014; Osorio-de-Castro et al., 2015; Souza et al., 2016; Saravu et al., 2018; Oduro et al., 2019; Rosa et al., 2020). The eligible studies assessed adherence to antimalarials prescribed for the treatment of malaria caused by *P. vivax* (10/21, 47.6%) and *P. falciparum* (16/21, 76.2%). The drugs for vivax malaria were chloroquine or ACT and primaquine, and treatment duration ranged from 7–14 days. In the studies on *falciparum* malaria, the treatment regimen prescribed was ACT, with a single exception (Fungladda et al., 1998).

**FIGURE 2:**
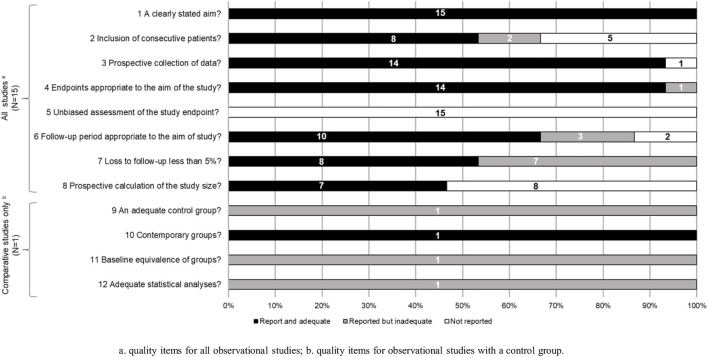
Quality assessment of the studies included in the systematic review according to the Methodological Index for Non-randomized Studies (Slim et al., 2003). **(A)**. quality items for all observational studies; **(B)**. quality items for observational studies with a control group.

**FIGURE 3:**
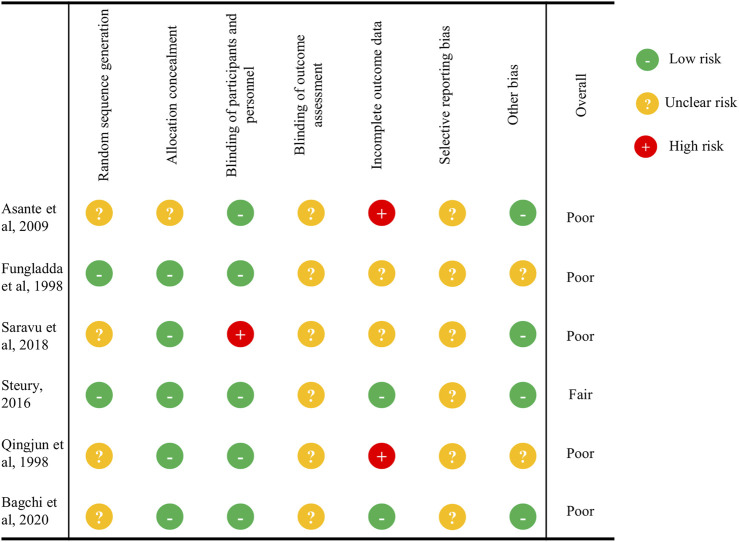
Quality assessment of the studies included in the systematic review according to the Cochrane Risk of Bias Tool for Randomized Controlled Trials for clinical trials (Higgins and Green, 2011).

The quality assessment of the studies is summarized in Supplementary Table S2 and Figure 2 and Figure 3. Most of the observational/quasi-experimental studies (13/15, 86.6%) collected data prospectively and reported appropriate endpoints. About half (8/15, 53.3%) of them reported adequately loss to follow up less than 5% and the prospective calculation of study size (7/15, 46.6%). The same was observed for the quasi-experimental study, which is the only one that has comparative groups. Of the six clinical trials included in this review, five were deemed to be poor quality (Fungladda et al., 1998; Qingjun et al., 1998; Asante et al., 2009; Saravu et al., 2018; Bagchi et al., 2020), and one fair quality (Steury, 2016) according to the Cochrane Risk of Bias tool. None of the trials reported whether blinding was used either when assigning patients to treatment arms or when measuring endpoints.

Quantitative estimates of antimalarial adherence varied among treatments and methods used to assess adherence (Supplementary Table S2). Estimates of the rate of adherence to *P. vivax* treatment differed considerably depending upon the method used to measure adherence. When adherence was measured based on a biological assay, the estimated adherence rate was 95.3% (Cheoymang et al., 2015) versus 71.1–100% based on pill counts (Almeida et al., 2014; Cheoymang et al., 2015; Osorio-de-Castro et al., 2015), 50–100% (Rocha, 2008; Cheoymang et al., 2015; Osorio-de-Castro et al., 2015) based on interviews, and 63.8–83% based on questionnaires (Qingjun et al., 1998; Almeida et al., 2014). When adherence was measured using a combination of different methods, the estimated rate of adherence varied from 44.1 to 75% (Souza et al., 2016; Saravu et al., 2018; Rosa et al., 2020). Studies of *P. falciparum* treatment also reported a wide range of adherence rates: 45.4–92.6% by pill count (Asante et al., 2009; Amponsah et al., 2015; Osorio-de-Castro et al., 2015), 66.7–100% by interview (Rocha, 2008; Minzi et al., 2014; Osorio-de-Castro et al., 2015; Takahashi et al., 2018), 16.7% by electronic pillbox (Steury, 2016), and 86.8–100% by biological assays (Na-Bangchang et al., 1997; Minzi et al., 2014). The rate of adherence based on a combination of methods varied from 60 to 94.4% (Takahashi et al., 2018; Oduro et al., 2019; Bagchi et al., 2020).

The studies included in the review assessed adherence by indirect and direct methods. The indirect methods described were self-reports, pill counts, MEMS, and clinical cure. To measure adherence to antimalarial treatment, 76.2% (16/21) of the studies used a combination of methods, the most frequent of which were self-reported adherence and pill counts (Fungladda et al., 1998; Lemma et al., 2011; Tun et al., 2012; Almeida et al., 2014; Ferreira et al., 2014; Osorio-de-Castro et al., 2015; Souza et al., 2016; Saravu et al., 2018; Takahashi et al., 2018; Oduro et al., 2019; Bagchi et al., 2020) (Supplementary Table S2 and Table 1).

**TABLE 1:** Description of the methods to assess adherence used on the included studies.

Author, Year	Adherence methods	Adherence methods description
Na-Bangchang et al. (1997)	Biological Assay	Blood Mefloquine concentrations on day 2 compared with reference profiles from hospitalized patients under supervisioned treament
Fungladda et al. (1998)	1. Self-reported Measure	1. Interview on day 5 for AS and day 7 for QN+TET^*^
	2. Pill count	2. The blister pack was examined on day 5 for AS and day 7 for QN+TET for remaining tablets
Qingjun et al. (1998)	Self-reported Measure	Questionnaire applied on day 8^*^
Fogg et al. (2004)	1. Self-reported Measure	1. Open questionnarie - a structured interview concerning the time and method of taking each dose, applied on day 3
	2. Pill count	2. The blister pack was examined on day 3 for remaining tablets
	3. Biological assay	3. Blood Lumefantrine concentrations correlated with the results of indirect methods assessed^*^
Rocha (2008)	1. Self-reported Measure	1. Interview asking if the participant took the medication as prescribed by the healthcare professional and describing how it was taken applied on day 7
	2. Clinical cure	2. Absence of symptoms assessed on day 7
Asante et al. (2009)	Pill count	The blister pack was examined on day 2 for remaining tablets
Lemma et al. (2011)	1. Self-reported Measure	1. Questionnaire applied on day 3^*^
	2. Pill count	2. The blister pack was examined on day 3 for remaining tablets
Tun et al. (2012)	1. Self-reported Measure	1. Questionnaire applied on day 3^*^
	2. Pill count	2. The blister pack was examined on day 3 for remaining tablets
Almeida et al. (2014)	1. Self-reported Measure	1. A 5 item self-reported questionnarie adding one question to Morisky’s 4-item questionnaire (Dichotomous and Likert scale) applied on day 7
	2. Pill count	2. The blister pack was examined on day 7 for remaining tablets
Ferreira et al. (2014)	1. Self-reported Measure	1. Interview with one question - “Could you take the prescribed medications?” applied on day 3 for *Pf* and day 6 for *Pv*
	2. Pill count	2. The blister pack was examined on day 3 for *Pf* and day 6 for *Pv* for remaining tablets
Minzi et al. (2014)	1. Self-reported Measure	1. Interview - A structured interview to determine how the regimen was taken, the time and method of taking each dose was then conducted, applied on day 3
	2. Pill count	2. The blister pack was examined on day 3 for remaining tablets
	3. Biological Assay	3. Blood Lumefantrine concentrations on day 7, that corresponds to 24 hours after 7 days of AL intake
Amponsah (2015)	Pill count	The blister pack was examined on day 3 for remaining tablets
Cheoymang et al. (2015)	1. Self-reported Measure	1. Interview without questionnaire applied on days 3, 7, and 14^*^
	2. Pill count	2. The blister pack was examined on days 3, 7, and 14 for remaining tablets
	3. Biological assay	3. Blood Primquine concentrations collected about 2–4 h after dosing on days 3, 7, and 14 of the initial treatment for the determination of primaquine concentrations, describing the minimum, maximum and outliers of plasma concentrations
Osorio-de-Castro et al. (2015)	1. Self-reported Measure	1. Interview applied on day 2 for *Pf* and day 5 for *Pv* ^*^
	2. Pill count	2. The blister pack was examined on day 2 for *Pf* and day 5 for *Pv* for remaining tablets
Souza et al. (2016)	1. Self-reported Measure	1. Interview with one question: “Could you take the prescribed medications?”
	2. Pill count	2. The blister pack was examined for remaining tablets
Steury (2016)	1. MEMS	1. The MEMS cap on the pillbox containing the ACT electronically recorded the time of each opening of the medication bottle beginning with the first dose on day 3 to 1 week
	2. Pill count	2. The blister pack was examined on day 3 to 1 week for remaining tablets
Saravu et al. (2018)	1. Self-reported Measure	1. Interview applied on day 6^*^
	2. Pill count	2. The blister pack was examined on day 6 for remaining tablets
Takahashi et al. (2018)	1. Self-reported Measure	1. Interview (home visit or telephone) applied on day 3 or 4^*^
	2. Pill count	2. The blister pack was examined on day 3 or 4 for remaining tablets
Oduro et al. (2019)	1. Self-reported Measure	1. Interview - The in-depth interview included a day-by-day account of the number of doses taken, number of tablets taken during each dose, time of each dose, reasons for any leftover or missed dose, and whether or not there was vomiting, applied on day 3
	2. Pill count	2. The blister pack was examined on day 3 for remaining tablets
Bagchi et al. (2020)	1. Self-reported Measure	1. Interview applied on day 3^*^
	2. Self-reported Measure	2. Subject’s self-reported compliance asked on day 3^*^
	3. Pill count	3. The blister pack was examined on day 3 for remaining tablets
Rosa et al. (2020)	1. Self-reported Measure	1. Morisky Medication Adherence Scale (MMAS-8) questionnaire

* The authors did not provide brief a description of the method. ACT, Artemisinin-based Combination Therapy; AL, Artemether + Lumefantrine; AS, Artesunate; MEMS, Medication Event Monitoring System; *Pf*, Plasmodium falciparum; *Pv*, Plasmodium vivax; QN, Quinine; TET, Tetracycline.

Five studies included detailed descriptions of the questionnaires/interviews (Rocha, 2008; Almeida et al., 2014; Ferreira et al., 2014; Souza et al., 2016; Rosa et al., 2020). Two studies used a single question to assess adherence. The question was “Could you take the prescribed medications?” and patients who answered “yes” were considered adherent and those responding “no”, non-adherent (Ferreira et al., 2014; Souza et al., 2016). Another study (Almeida et al., 2014) developed a 5-item questionnaire by adding the following question to Morisky’s 4-item instrument (Morisky et al., 1986): “Do you replicate the dose when you are feeling sick?” The patients’ responses to this question were evaluated using both a dichotomous yes/no scale and a Likert scale (“all the time”, “nearly always”, “usually”, “sometimes”, “once a while”, and “never”). One study used the Morisky Medication Adherence Scale 8-item (MMAS-8) (Morisky et al., 2008) questionnaire with dichotomous responses (Rosa et al., 2020); however, study did not include a definition of adherence. Seven studies (Fogg et al., 2004; Lemma et al., 2011; Tun et al., 2012; Minzi et al., 2014; Souza et al., 2016; Oduro et al., 2019; Bagchi et al., 2020) reported that the questionnaire applied included the time and date of the medication used by the patient (Table 2).

**TABLE 2:** Description of adherence categories.

Method	Categories	Definition	Study
Self-reports	Adherent	Report of taking the medicines as prescribed	Rocha, (2008)
	Adherent	values > median*	Almeida et al. (2014)
	Adherent	Report no missed doses during treatment period	Osorio-de-Castro et al. (2015)
Self-report and pill count	Adherent	adherent report and no tablets remaining	Fungladda et al. (1998)
	Definitely non-adherent	tablets remaining	Fogg et al. (2004), Lemma et al. (2011), Tun et al. (2012)
	Probably non-adherent	empty or missing blister and report not following the scheme (taking all doses at the correct time on the correct day and correct amount)	
	Probably adherent	empty or missing blister and report following the scheme (taking all doses at the correct time on the correct day and correct amount)	
	Adherent	answered “yes” and 100% pills taken of CQ, and 70% pills taken of PQ or 70% pills taken of AL	Ferreira et al. (2014)
	Definitely non-adherent	Tablets unfinished	Minzi et al. (2014)
	Probably non-adherent	empty or missing blister and wrong dose/incorrect time	
	Probably adherent	empty or missing blister and correct dose/correct time	
	Definitely adherent	no tablets remaining and correct dose/correct time	
	Definitely non-adherent	tablets remaining	Takahashi et al. (2018)
	Probably non-adherent	empty or missing blister and the patient answered “having not taken all doses”.	
	Probably adherent	empty or missing blister and the patient answered “having taken all of the doses.”	
	Probably non-adherent	if the patient answered “having not taken all doses”. – telephone	
	Probably adherent	if the patient answered “having taken all of the doses” and “taken on each day of the regimen.” – telephone	
	Complete adherence	reported taking all doses as recommended and no pill left in the pack.	Oduro et al., (2019)
	Incomplete adherence	reported that they did not take all the doses as recommended and a greater than or less than the expected number of pills.	
	Definitely non-adherent	did not take the tablets at all or as recommended and a greater than expected number of pills	
	Adherent	when all the doses of study medications were taken at the correct time on the correct day and in the correct amount.	Bagchi et al. (2020)
	Non-adherent	if tablets remained in the blister pack or when reporting inadequate intake of dose and/or timing of tablets	
Pill count	Adherent	>70% of pills taken	Almeida et al. (2014)
	Fully adherent	100% of pills taken	Amponsah et al. (2015)
	Partially adherent	70–<100% of pills taken	
	Non-adherent	<70% of pills taken	
	Adherent	quantity received as proxy of quantity consumed	Osorio-de-Castro et al. (2015)
	Adherent	no medication tablets remaining report following the scheme (taking all doses at the correct time on the correct day and correct amount)	Souza et al. (2016)
	Non-adherent	remaining medication tablets or stated any irregularity in adherence to the treatment regimen	
Biological assay	Fully adherent	concentrations within or above reference interval of MQ (1587-2572 µg/L)	Na-Bangchang et al. (1997)
	Partially adherent	concentrations bellow reference interval of MQ (1587-2572 µg/L)	
	Non-adherent	concentrations undetectable	
	Adherent	concentration of Lumefantrine ≥175 ng/mL	Minzi et al. (2014)
MEMS and pill count	Probably adherent	recorded bottle opening times according to the designated ranges (bottle opening within 1 hour of the prescribed time for the second dose (8 h after initial dose), and a recorded bottle opening within 2 h of the prescribed time for the next 2 days’ doses (8 a.m. and 8 p.m. on each day) and no tablets remaining	Steury, (2016)
	Probably non-adherent	requirement was not satisfied	
MEMS	Probably perfectly adherent	as defined by digitally recorded MEMS bottle opening occurring only during the time frames of adherence and only the required six openings with one in each time frame	Steury, (2016)
Clinical cure	Adherent	Absence of symptoms on the assessment day	Rocha, (2008)

* Likert scale (LS) was dichotomized (LDS) and grouped with the Dichotomous scale (DS) into an overall dichotomous scale (ODS). LS were determined by the sum of the percentage of each item divided by the total of the item, and in the DS by simply adding each item. AL, Artemether + Lumefantrine; CQ, Chloroquine; MEMS, Medication Event Monitoring System; MQ, Mefloquine; PQ, Primaquine; *Pf*, Plasmodium falciparum; *Pv*, Plasmodium vivax.

More than half of the studies that utilized pill counts (12/18, 66.6%) classified the patient as adherent if there were no tablets remaining upon study completion (Fungladda et al., 1998; Fogg et al., 2004; Lemma et al., 2011; Tun et al., 2012; Minzi et al., 2014; Amponsah et al., 2015; Osorio-de-Castro et al., 2015; Souza et al., 2016; Steury, 2016; Takahashi et al., 2018; Oduro et al., 2019; Bagchi et al., 2020). Four studies compared self-reports to pill counts as a validatory step. In Fogg et al.’s (2004) and Osorio-de-Castro et al.’s (2015) studies, there was a substantial concordance between methods (Kappa coefficients 0.81 and 0.74). In Almeida et al.’s (Almeida et al., 2014) and Minzi et al.’s (2014) studies, the concordance was almost perfect (Kappa 0.94 and 0.96).

Adherence was measured directly by biological assays quantifying lumefantrine (Fogg et al., 2004; Minzi et al., 2014), mefloquine (Na-Bangchang et al., 1997), and primaquine (Cheoymang et al., 2015) in blood. The assessment required defining a threshold concentration above which the patient was considered adherent. The reference threshold varied among studies. In one study, the threshold was the median concentration of antimalarials in hospitalized patients (Na-Bangchang et al., 1997). Another utilized a pre-established concentration from a previous study (Minzi et al., 2014). The two remaining studies utilized a different method to determine the threshold (Fogg et al., 2004; Cheoymang et al., 2015). Fogg et al. (2004) correlates lumefantrine concentrations with the results of indirect methods assessed; Cheoymang et al. (2015) describes minimum, maximum and outliers primaquine plasma concentrations (Table 1).

The timing of the assessment of adherence differed somewhat among the studies. Most studies (17/21, 80.9%) measured adherence 1 day after treatment ended and two studies measured it on the final day of treatment (Na-Bangchang et al., 1997; Asante et al., 2009). In other two studies, adherence was assessed more than once (Minzi et al., 2014; Cheoymang et al., 2015). Finally, a single study (Osorio-de-Castro et al., 2015) evaluated adherence in the course of treatment. Furthermore, the articles utilized distinct models for following up with patient’s post-treatment. In the majority of studies (11/21, 52.4%), follow-up took the form of home visits (Fogg et al., 2004; Asante et al., 2009; Lemma et al., 2011; Tun et al., 2012; Almeida et al., 2014; Ferreira et al., 2014; Minzi et al., 2014; Osorio-de-Castro et al., 2015; Souza et al., 2016; Takahashi et al., 2018; Oduro et al., 2019). In six studies, the patient was required to return to the clinic (Na-Bangchang et al., 1997; Fungladda et al., 1998; Rocha, 2008; Cheoymang et al., 2015; Steury, 2016; Saravu et al., 2018), whereas three studies did not report where the follow up took place (Amponsah et al., 2015; Bagchi et al., 2020; Rosa et al., 2020). One study reported the use of telephone interview when home visits could not be conducted (Takahashi et al., 2018) and another used both telephone interviews and home visits (Qingjun et al., 1998) (Supplementary Table S2 and Table 1).

In total, sixteen studies classified adherence into categories. We identified five distinct systems for classifying adherence among these studies. In seven studies, participants were classified as “adherent” or “non-adherent” (Fungladda et al., 1998; Rocha, 2008; Almeida et al., 2014; Ferreira et al., 2014; Osorio-de-Castro et al., 2015; Souza et al., 2016; Bagchi et al., 2020); two studies used “fully adherent”, “partially adherent” and “non-adherent” (Na-Bangchang et al., 1997; Amponsah et al., 2015), five used “definitely non-adherent”, “probably non-adherent”, “probably adherent” (Fogg et al., 2004; Lemma et al., 2011; Tun et al., 2012; Minzi et al., 2014; Takahashi et al., 2018), one study used “probably perfectly adherent”, “probably adherent”, “probably non adherent”, and “probably not perfectly adherent” (Steury, 2016), and another study, which was the only study that utilized MEMS, classified participants as “complete adherent”, “incomplete adherent”, and “definitely non-adherent” (Oduro et al., 2019) (Supplementary Table S2). Twelve studies (Fungladda et al., 1998; Fogg et al., 2004; Lemma et al., 2011; Tun et al., 2012; Ferreira et al., 2014; Minzi et al., 2014; Souza et al., 2016; Steury, 2016; Saravu et al., 2018; Oduro et al., 2019; Bagchi et al., 2020; Rosa et al., 2020) used a combination of methods to classify the adherence (Table 2).

## Discussion

We reviewed a variety of methods for assessing adherence to antimalarials among patients whose infection was confirmed by parasitological examination. More than half of the studies (14/21, 66.7%) were published in the past decade, and one third (7/21, 33.3%) in the last 5 years, suggesting that concern about adherence has increased. Irrespective of the malaria species or drug regimen, the most frequently used methods to measure adherence were pill counts and self-reports. The widespread use of these methods can be attributed to their low cost (Gabarró, 1999), straightforward implementation, and suitability for any therapeutic regimen.

In eleven studies that used pill counts, home visits were realized to increase follow-up (Qingjun et al., 1998; Fogg et al., 2004; Asante et al., 2009; Lemma et al., 2011; Tun et al., 2012; Almeida et al., 2014; Minzi et al., 2014; Osorio-de-Castro et al., 2015; Souza et al., 2016; Takahashi et al., 2018; Oduro et al., 2019). Similar to studies of chronic diseases, most of these studies defined adherence as consumption of 70% of pills (Almeida et al., 2014; Ferreira et al., 2014; Amponsah et al., 2015). This cut-off may not be suitable for the treatment of an acute infectious disease like malaria, where the goal has to be completing a full therapeutic scheme. A limitation of pill counts is that the counts might not provide information on either the timing of consumption or reasons for non-adherence (Krousel-wood et al., 2004; Lam and Fresco, 2015). Furthermore, it is impossible to confirm whether missing pills were ingested rather than lost or discarded (social desirability bias). This bias can be reduced using unannounced visits, as performed by the studies of Fogg et al. (2004) and Minzi et al. (2014).

Self-reported methods differ in complexity. Interviews and questionnaires examine behavior, beliefs, attitudes toward symptoms, and the patient’s understanding of the drug regimen. Reasons for non-adherence reported in the literature include forgetfulness, adverse reactions, misunderstanding of medication instructions, and the patient’s belief of cure before the end of treatment (Fungladda et al., 1998; Fogg et al., 2004; Lemma et al., 2011; Ferreira et al., 2014; Amponsah et al., 2015; Cheoymang et al., 2015). However, self-reported reasons for non-adherence are subject to recall and social desirability bias if the patient deliberately or unintentionally withholds information (Garber et al., 2004; Cook et al., 2005). As methods of self-reporting varied among studies, it is difficult to assess the reliability between measures.

In the studies included in this review, MEMS and clinical cure were indirect methods that were always used together with other methods (Rocha, 2008; Steury, 2016). Electronic pillboxes are capable of recording the date and time when the bottle was opened, making it possible to recognize patterns of medication use such as only opening the pillbox before the follow-up visits (“White Coat Adherence”) (Schwed et al., 1999; Ailinger et al., 2008). However, pillbox opening does not guarantee ingestion of the pills and, just as failure to open the pillbox does not mean the pills are not being taken. Due to their high cost, the use of electronic pillboxes has been restricted primarily to clinical trials involving a single drug. This tends to limit the utility of MEMS to malaria monotherapy. When electronic pillboxes cannot be used, adherence can be measured via self-reports, pill counts, or biological assays, with blinding of the possible follow up visit to minimize “White Coat Adherence” (Fogg et al., 2004; Minzi et al., 2014).

Clinical cure was defined as the absence of malaria symptoms 6 days after diagnosis (Rocha, 2008). A study in Brazil reported that although symptoms of vivax malaria disappeared on the second day of treatment, 22.5% of patients still had a positive smear (Abdon et al., 2001). This finding casts doubt on the accuracy of clinical cure as an indicator of adherence. In light of this, clinical cure should be combined with laboratory confirmation of cure.

Less than a quarter of the studies (4/21, 23.5%) used direct methods that measured drug concentrations in blood (Na-Bangchang et al., 1997; Fogg et al., 2004; Minzi et al., 2014; Cheoymang et al., 2015), which provide the strongest evidence that the patient ingested the medication (Farmer, 1999; de Achaval and Suarez-Almazor, 2010; Bemt et al., 2012; Lam and Fresco, 2015). This is likely due to the fact that direct methods require specialized training and laboratory resources making them expensive and invasive. Furthermore, drug interactions and variations in drug pharmacokinetics may interfere with the evaluation of these methods. For instance, changes in the CYP 2D62C8 metabolic pathways can increase the risk of therapeutic failure of primaquine (Ingelman-Sundberg, 2005) and modify the kinetics of chloroquine (Kim et al., 2003), altering the perception of adherence. In addition, in the study of Fogg et al. (2004), the plasma concentration of lumefantrine was not used to classify adherence because the fraction absorbed is highly variable, and it was used only to assess the correlation with the indirect methods. These combination of factors impact on the feasibility of direct methods in clinical practice (Lam and Fresco, 2015). Furthermore, the definition of the reference value of drug concentrations is a challenge. None of the studies included utilized the same reference value. Half of them (2/4, 50%) (Fogg et al., 2004; Cheoymang et al., 2015) calculated the mean concentration of the adherent and non-adherent patients. As treatments for malaria are based on combined therapy avoid the evolution of resistance, the assessment of adherence via biological assays should consider all of the drugs that are included in the treatment regimen.

The timing of assessment of adherence was appropriate in all included studies. The proximity of self-report to the completion of treatment is beneficial as it tends to reduce recall problems. In studies of MEMS, there was no risk of memory bias since the date and time when the pill bottle was opened were recorded electronically.

We found that rates of medication adherence were classified into a wide variety of qualitative categories, which were not standardized across the studies. While seven of the 21 studies adopted binary classification as “adherent” or “non-adherent” (Fungladda et al., 1998; Rocha, 2008; Almeida et al., 2014; Ferreira et al., 2014; Osorio-de-Castro et al., 2015; Souza et al., 2016; Bagchi et al., 2020), four other categorizations were also used in the studies included in this review (Na-Bangchang et al., 1997; Fogg et al., 2004; Lemma et al., 2011; Tun et al., 2012; Minzi et al., 2014; Amponsah et al., 2015; Steury, 2016; Takahashi et al., 2018; Oduro et al., 2019). Recent studies have described adherence as a “spectrum” of behaviors ranging from refusal of treatment, to partial conformity, to precisely following the prescription (Julius et al., 2009). Until standardized categories are adopted in the literature, it will remain difficult to compare data from different studies and assess the efficacy of adherence-increasing interventions.

In our view it is beneficial to use multiple, complementary techniques, as any given method for measuring adherence will have limitations. A high level of concordance between pill count and self-report methods was shown in four studies included in this review (Fogg et al., 2004; Almeida et al., 2014; Minzi et al., 2014; Osorio-de-Castro et al., 2015). However, only two studies (Fogg et al., 2004; Minzi et al., 2014) used announced visits to minimize the social desirability bias, common in both methods. It should be noted that subjective and objective methods assess different dimensions of adherence. While subjective methods are useful in ascertaining the beliefs or barriers to adherence, objective methods provide more accurate data on the way patients intake in their medication regimens. As a gold standard method is not currently available, like other authors (Brown et al., 2016; Anghel et al., 2019), we recommend that two or three approaches be used in parallel. Since the resources available for antimalarial treatment may vary considerably among treatment sites, the best method to be applied in one setting may not necessarily be the best in another. Methods that require specialized equipment and personnel, such as biological assays and MEMS, tend to be more difficult to apply in clinical practice than in research settings, while other indirect methods can be applied in both.

The duration, timing, and frequency of doses were reported in eight studies included in this review (Fogg et al., 2004; Lemma et al., 2011; Tun et al., 2012; Minzi et al., 2014; Souza et al., 2016; Steury, 2016; Oduro et al., 2019; Bagchi et al., 2020). We recommend that these variables should be measured whenever possible. The study that used MEMS (Steury, 2016) detected lower adherence (16.7%) than the others. As electronic pillboxes automatically record the timing and frequency of doses, they are able to detect suboptimal adherence with high sensitivity (El Alili et al., 2016).

Among the strengths of this review are that we searched eight databases and the grey literature, with no restrictions on language, publication date, or drug regimen and only included studies with confirmed parasitological diagnosis. The limitations include the lack of information about the instruments used for self-reports, and uncertainty about medication intake based on pill counts and the cut-off value for biological assays. Another potential weakness of this review is that we did not request unpublished data from the authors of the eligible studies.

Future studies about adherence to antimalarial treatment should describe their methods in sufficient detail so that they can be replicated and utilize standardized categories of adherence to facilitate comparisons. Clinical outcomes such as clinical and radical cure can be used to define cut-off points that optimally stratifies the good versus poor adherence categories (Karve et al., 2009; O’Halloran Leach et al., 2021). A validation step should be considered for the methods, mainly for the indirect ones, but not for the direct adherence markers. For new instruments to be developed, self-reports should measure whether the patient was able to take the medications as prescribed, several times during the follow-up to test consistency in the response. Further, self-reports should assess if the patient ever missed a dose or experienced adverse events, as these data can be used to improve future therapies. Another suggestion is the development of studies that combine direct and indirect methods as a way to validate the different types of self-reports (Wilson et al., 2016). Studies designed to determine a range of cut-off values to assess drug metabolism should also be performed. The DOT can be used with direct methods for the definition of malaria drug concentration threshold, as well as assist in the development and validation of point -of- care tests to assess adherence (Gandhi et al., 2019). It is important to consider that using methods developed for chronic diseases might not be suitable for an acute disease like malaria, since the consequences of non-adherence are different. In antimalarial treatment, suboptimal medication adherence can cause relapses, severe malaria, death, development of antimalarial resistance, and spread of the disease (Duarte and Gyorkos, 2003; Bruxvoort et al., 2014; Siddiqui et al., 2015; World Health Organization and Global Malaria Programme, 2015).

Indirect methods for assessing adherence to antimalarial treatment have been used more frequently than direct ones and seem to be the most practical, irrespective of the malaria species or therapeutic scheme. In our view, the best approach for measuring treatment adherence is a combination of methods that evaluate adherence using different parameters and are feasible given local resources. Combining an objective method that gives solid proof of the ingestion of medication and a subjective method that complements the research with information regarding factors, beliefs or barrier of adherence seems to be the best approach. There is a need for methods that combine these approaches in a cost -effectiveness way.

Our review underscores the importance of developing an optimum adherence classification by methods and validating methods for assessing adherence to antimalarial treatment, including specific method for evaluating the causes of non-adherence, as it is a fundamental tool for improving the efficacy of therapy and control.

## Data Availability

The raw data supporting the conclusions of this article will be made available by the authors, without undue reservation.

## Abbreviations

AS, Artesunate; AQ, Amodiaquine; AL, Artemether + Lumefantrine; AM, Arthemeter; MQ, Mefloquine; CQ, Chloroquine; DHA, Dihidroartemisinine; DOX, Doxycycline; G6PD, Glucose 6 Phosphate Dehydrogenase; DOT, Directly Observed Therapy, MEMS, Medication Event Monitoring System; MINORS, Methodological Index for Non-randomized Studies; PIP, Piperaquine; PQ, Primaquine; Pf, Plasmodium Falciparum; Pv, Plasmodium vivax; QN, Quinine; TET, Tetracycline; WHO, World Health Organization.
