# Supplementary material for: Methods to Assess Adult and Adolescent Patients’ Adherence to Antimalarial Treatment: A Systematic Review
Source: Front Pharmacol. 2022 Apr 27;13:796027. doi: 10.3389/fphar.2022.796027 (PMC9092497; doi:10.3389/fphar.2022.796027)
Supplement: Supplementary file 2 [file Table1.docx]

**Supplementary Table S1**. Search strategy

| Database | Search Strategy |
| --- | --- |
| Medline | ((((((anti malarials[MeSH Terms]) OR anti malarials[Title/Abstract])) AND (((("malaria"[MeSH Terms]) OR "malaria"[Title])) AND ((drug therapy[MeSH Terms]) OR treat*[Title])))) AND ((((((((((adherence, medication[MeSH Terms]) OR adherence, patient[MeSH Terms]) OR compliance, medication[MeSH Terms]) OR compliance, patient[MeSH Terms]) OR non adherence, medication[MeSH Terms]) OR non adherence, patient[MeSH Terms]) OR non compliance, medication[MeSH Terms]) OR non compliance, patient[MeSH Terms])) OR adherence[Title])) AND "humans" |
| WOS | (((TOPIC: (malaria) OR TITLE: (malaria)) AND (TITLE: (treat$) OR TOPIC: (therapy) OR TOPIC: (drug therapy))) AND (TOPIC: (adherence patient) OR TOPIC: (adherence medication) OR TOPIC: (patient compliance) OR TOPIC: (medication compliance) OR TITLE: (adherence) OR TITLE: (compliance))) |
| Embase | ('malaria'/de OR 'malaria':ti) AND ('therapy'/de OR 'therapy':ti OR 'drug therapy'/de OR 'drug therapy':ti) AND ('patient compliance'/de OR 'patient compliance':ti OR 'medication compliance'/de OR 'medication compliance':ti OR 'adherence':ti) AND ('human'/exp OR human) |
| Scopus | ( KEY( "malaria") OR TITLE("malaria”) ) AND ( KEY ( "adherence, medication" ) OR KEY ( "adherence, patient" ) OR KEY ( "compliance, medication" ) OR KEY ( "compliance, patient" ) OR TITLE ( "adherence, patient" ) OR TITLE ( "adherence, medication" ) OR TITLE ( "compliance, patient" ) OR TITLE ( "compliance, medication" ) OR TITLE ( "compliance" ) OR TITLE ( "adherence" ) ) |
| Lilacs | (((((mh:(malaria)) OR (ti:(malaria))) AND ((mh:(therapy)) OR (ti:(treatment)))) OR (mh:(antimalarials)) OR (ti:(antimalarials))) AND ((mh:(medication adherence)) OR (mh:(patient compliance)) OR (ti:(patient compliance)) OR (ti:(medication adherence)) OR (ti:(adherence)) OR (ti:(compliance)) OR (mh:(non adherence, patient )) OR (mh:(non compliance, patient)))) |
| Scholar | allintitle: malaria treatment adherence OR compliance |
| Cochrane | ((("malaria"):ti,ab,kw AND ("treatment":ti,ab,kw or "therapy":ti,ab,kw)) OR ("antimalarial":ti,ab,kw))) AND ((adherence, medication:ti,ab,kw or adherence, patient:ti,ab,kw or compliance, patient:ti,ab,kw or compliance, medication:ti,ab,kw or "adherence":ti) or (“compliance”:ti)) |
| Greynet | Malaria adherence  Malaria compliance |
