# Supplementary material for: Methods to Assess Adult and Adolescent Patients’ Adherence to Antimalarial Treatment: A Systematic Review
Source: Front Pharmacol. 2022 Apr 27;13:796027. doi: 10.3389/fphar.2022.796027 (PMC9092497; doi:10.3389/fphar.2022.796027)
Supplement: Supplementary file 3 [file Table2.docx]

| Table 2. Description of adherence categories | | | |
| --- | --- | --- | --- |
| Method | **Categories** | **Definition** | **Study** |
| Self-reports | Adherent | Report of taking the medicines as prescribed | Rocha, 2008 |
|  | Adherent | values > median* | Almeida et al, 2014 |
|  | Adherent | Report no missed doses during treatment period | Osorio-de-Castro et al, 2015 |
| Self-report and pill count | Adherent | adherent report and no tablets remaining | Fungladda et al, 1998 |
|  | Definitely non-adherent | tablets remaining | Fogg et al, 2004; Lemma et al, 2011; Tun et al, 2012 |
|  | Probably non-adherent | empty or missing blister and report not following the scheme (taking all doses at the correct time on the correct day and correct amount) |  |
|  | Probably adherent | empty or missing blister and report following the scheme (taking all doses at the correct time on the correct day and correct amount) |  |
|  | Adherent | answered "yes" and 100% pills taken of CQ, and 70% pills taken of PQ or 70% pills taken of AL | Ferreira et al, 2014 |
|  | Definitely non-adherent | Tablets unfinished | Minzi et al, 2014 |
|  | Probably non-adherent | empty or missing blister and wrong dose/incorrect time |  |
|  | Probably adherent | empty or missing blister and correct dose/correct time |  |
|  | Definitely adherent | no tablets remaing and correct dose/correct time |  |
|  | Definitely non-adherent | tablets remaining | Takahashi et al, 2018 |
|  | Probably non-adherent | empty or missing blister and the patient answered “having not taken all doses”. |  |
|  | Probably adherent | empty or missing blister and the patient answered “having taken all of the doses.” |  |
|  | Probably non-adherent | if the patient answered “having not taken all doses”. - telephone |  |
|  | Probably adherent | if the patient answered “having taken all of the doses” and “taken on each day of the regimen.” - telephone |  |
|  | Complete adherence | reported taking all doses as recommended and no pill left in the pack. | Oduro et al, 2019 |
|  | Incomplete adherence | reported that they did not take all the doses as recommended and a greater than or less than the expected number of pills. |  |
|  | Definitely non-adherent | did not take the tablets at all or as recommended and a greater than expected number of pills |  |
|  | Adherent | when all the doses of study medications were taken at the correct time on the correct day and in the correct amount. | Bagchi et al, 2020 |
|  | Non-adherent | if tablets remained in the blister pack or when reporting inadequate intake of dose and/or timing of tablets |  |
| Pill count | Adherent | >70% of pills taken | Almeida et al, 2014 |
|  | Fully adherent | 100% of pills taken | Amponsah, 2015 |
|  | Partially adherent | 70-<100% of pills taken |  |
|  | Non-adherent | <70% of pills taken |  |
|  | Adherent | quantity received as proxy of quantity consumed | Osorio-de-Castro et al, 2015 |
|  | Adherent | no medication tablets remaining report following the scheme (taking all doses at the correct time on the correct day and correct amount) | Souza et al, 2016 |
|  | Non-adherent | remaining medication tablets or stated any irregularity in adherence to the treatment regimen |  |
| Biological assay | Fully adherent | concentrations within or above reference interval of MQ (1587-2572 µg/L) | Na-Bangchang et al, 1997 |
|  | Partially adherent | concentrations bellow reference interval of MQ (1587-2572 µg/L) |  |
|  | Non-adherent | concentrations undetectable |  |
|  | Adherent | concentration of Lumefantrine ≥175ng/mL | Minzi et al, 2014 |
| MEMS and pill count | Probably adherent | recorded bottle opening times according to the designated ranges (bottle opening within 1 hour of the prescribed time for the second dose (8 hours after initial dose), and a recorded bottle opening within 2 hours of the prescribed time for the next 2 days’ doses (8 a.m. and 8 p.m. on each day) and no tablets remaining | Steury, 2016 |
|  | Probably non-adherent | requirement was not satisfied |  |
| MEMS | Probably perfectly adherent | as defined by digitally recorded MEMS bottle opening occurring only during the time frames of adherence and only the required six openings with one in each time frame | Steury, 2016 |
| Clinical cure | Adherent | Absence of symptoms on the assessment day | Rocha, 2008 |
| *Likert scale (LS) was dichotomized (LDS) and grouped with the Dichotomous scale (DS) into an overall dichotomous scale (ODS). LS were determined by the sum of the percentage of each item divided by the total of the item, and in the DS by simply adding each item. AL - Artemether + Lumefantrine; CQ - Chloroquine; MEMS - Medication Event Monitoring System; MQ - Mefloquine; PQ - Primaquine; *Pf* – Plasmodium falciparum; *Pv* – Plasmodium vivax. | | | |
